# Factors associated with diarrheal disease in the rural Caribbean region of Colombia

**DOI:** 10.11606/s1518-8787.2020054002054

**Published:** 2020-09-24

**Authors:** Maria-Angelica Galezzo, Wanda Maria Risso Günther, Fredi Alexander Diaz-Quijano, Manuel Rodriguez Susa

**Affiliations:** I Universidad de los Andes Centro de Investigaciones en Ingeniería Ambiental Departamento de Ingeniería Civil y Ambiental BogotáDC Colombia Universidad de los Andes. Centro de Investigaciones en Ingeniería Ambiental. Departamento de Ingeniería Civil y Ambiental. Bogotá, DC, Colombia; II Universidade de São Paulo Laboratório de Gestão Ambiental, Inovação e Sustentabilidade São PauloSP Brasil Universidade de São Paulo. Laboratório de Gestão Ambiental, Inovação e Sustentabilidade. São Paulo, SP, Brasil; III Universidade de São Paulo Faculdade de Saúde Pública Laboratório de Inferência Causal em Epidemiologia São PauloSP Brasil Universidade de São Paulo. Faculdade de Saúde Pública. Laboratório de Inferência Causal em Epidemiologia. São Paulo, SP, Brasil

**Keywords:** Diarrhea, Infantile, epidemiology, Diarrhea, epidemiology, Risk Factors, Socioeconomic Factors, Rural Sanitation, Rural Health

## Abstract

**OBJECTIVE:**

To analyze factors associated with diarrheal disease in the rural Caribbean region of Colombia.

**METHOD:**

A cross-sectional study conducted in the rural area of the Cesar Department, Colombia, between November 2017 and June 2018. Self-reported cases of diarrheal disease were surveyed, and water samples from 42 households were collected and analyzed. Descriptive statistics were employed in the analysis of socioeconomic status, environmental and sanitary conditions, and we evaluated their association with the diarrheal disease using the Poisson regression models. Each model was adjusted with variables suggested by specific directed acyclic graphs.

**RESULTS:**

Poor water supply conditions, hygiene and basic sanitation were reported in the study area. All water samples were classified either as high risk for health problems or unfit for human consumption. The diarrheal disease had a prevalence of 7.5% across all ages and of 23.5% in children under five years old. The variables rainy season (PR = 0.24; 95%CI 0.07–0.85), children under five years old (PR = 4.05; 95%CI 1.70–9.68), water from deep wells (PR = 16.90; 95%CI 2.45–116.67), water from artificial ponds (PR = 11.47; 95%CI 1.27–103.29), toilets availability (PRA = 0.23; 95%CI 0.06–0.96), and swine presence (PR = 0.20; 95%CI 0.05–0.74) were significantly associated with the occurrence of diarrheal disease.

**CONCLUSION:**

Water supply, hygiene and basic sanitation conditions have been associated with the diarrheal disease, affecting almost a quarter of the population under five years old. There is an urge for the design of effective policies that improve environmental and sanitation conditions in rural areas.

## INTRODUCTION

Low socioeconomic status ^1^^,^^2^ , poor basic sanitation ^2^^-^^4^ , unsafe water consumption ^3^^,^^4^ , poor hygiene habits ^2^^-^^5^ , keeping of domestic animals ^6^ , among other factors, make the Latin American, Asian and African rural areas prone to develop waterborne diseases. Diarrheal disease stands out, amounting to 525,000 deaths and 1.7 billion cases in children under five years old worldwide ^7^ .

Rural communities represent 83% of the population lacking access to an improved water source, and 71% of those lack basic sanitation. Fifty-seven per cent of the households located in the rural area of Colombia lack access to drinking water and 94% lack access to sewage systems; in the Cesar Department, such percentages increase up to 87 and 98%, respectively ^9^ .

Within the framework of the Sustainable Development Goals (ended in 2015), projects were created and implemented in Colombia, such as: *Red Unidos* (United Network) for the reduction of poverty, *Sistema para la Protección y Control de la Calidad del Agua* (Protection and Control of Water Quality System) for human consumption, and the Colombia National System of Public Health Surveillance, among others. Such projects contribute to the improvement of living conditions of the population. However, larger efforts are required in the elaboration of differential and intersectoral policies, which should be adjusted to the context of rural areas and contribute to reducing the current gap ^10^^,^^11^ , enabling greater equality and improvements in the health of rural populations.

For such purposes, it is essential do recognize key factors of the diarrheal disease in rural areas to propose solutions with larger sanitation and environmental reaches. To meet this need, we analyzed factors associated with diarrheal disease in the rural Caribbean region of Colombia.

## METHODS

This study was conducted in the rural district of La Delfina (10º 19' N, 73º 28' W) and El Cascajo (9° 25' N, 73° 57' W), located 40 and 194 km away, respectively, from Valledupar city, the capital of the Cesar Department. The study area was selected by prioritizing the municipalities using the Electre ^12^ and Topsis ^12^ methods. The selection criteria were: i) aqueduct coverage, ii) greater number of inhabitants, iii) greater child mortality rate (1998 – 2015), and iv) lower rate of global Multidimensional Poverty Index (MPI), which was previously weighted by the CRITIC ^13^ and Entropía ^14^ methods. Analyzed data were those of the rural area of each municipality.

Then we selected two rural districts (the smaller administrative unit) through an Analytic Hierarchy Process ^12^ , into which we engaged knowledgeable actors of the rural area from prioritized municipalities. The evaluated criteria were: i) shorter aqueduct coverage, ii) increased water scarcity, iii) ease of access, and iv) greater community participation.

The rural districts were censused in September 2017 to identify population size, sanitation conditions, and water supply for human consumption. A geographic information system (GIS) was created with the household coordinates. The results of the census were used to elaborate a tool for data collection and identify the type of sampling for water collection.

A questionnaire was elaborated based on the *Censo Nacional Agropecuario* (CNA – Agricultural National Census) from 2014 and other similar studies ^16^^,^^17^ , which were adjusted to the study area. The questionnaire comprised 68 questions related to demographic aspects of the population; socioeconomic status, sanitation and water supply, and storage conditions in the households. A pilot experiment was conducted prior to the interview process and adjustments were made.

In order to comprehend the dry and rainy seasons, monthly rates of precipitation (1985–2015) were extracted from weather stations close to the study areas. The interviews and sample collection were randomly performed in November 2017, and March and June 2018.

The interviews were conducted by a trained group (one doctoral student and three students holding a bachelor's degree in environmental engineering) with the household chief or a resident older than 18 years old. The water samples were collected from the storage containers in each household, according to the Colombian guidelines. Analysis of the physical, chemical and biological parameters was carried out in the Environmental Engineering Laboratory of Los Andes University.

Besides household conditions, there was a survey on the occurrence of diarrheal cases within seven days before the visit. Pre-existing conditions were inquired to prevent interferences in the identification of cases of diarrheal disease: pregnancy, stomach surgery, gastric ulcers or any colon-related disease.

To classify the water quality, we used the *Índice de Riesgo de la Calidad del Agua para Consumo Humano* (IRCA – Water Quality Risk for Human Consumption Index), according to the guidelines of the Resolution No. 2115, from 2007 ^19^ . The IRCA values range from 0 to 100 and are classified according to the risk they pose to human consumption: no risk (≤ 5), low risk (5.1 to 14), medium risk (14.1 to 35), high risk (35.1-80) and unsanitary for human consumption (80.1-100).

The diarrheal disease was the dependent variable, and it had been defined as the occurrence of three or more loose stools within a 24-hour window. The prevalence of the disease within the seven days before data collection was estimated as the proportion of residents that had presented at least one event in the seven-day period before the interview.

The socioeconomic, basic sanitation and water supply conditions in the household, along with the demographic conditions collected were exposure variables.

The absence of poultry in only two households absolutely ruled out the occurrence of the event, given that all cases of diarrheal disease had occurred in households keeping poultry. Thus, this variable had not been included in the model due to the impossibility of getting adjusted measures of association.

**Figure 1:**
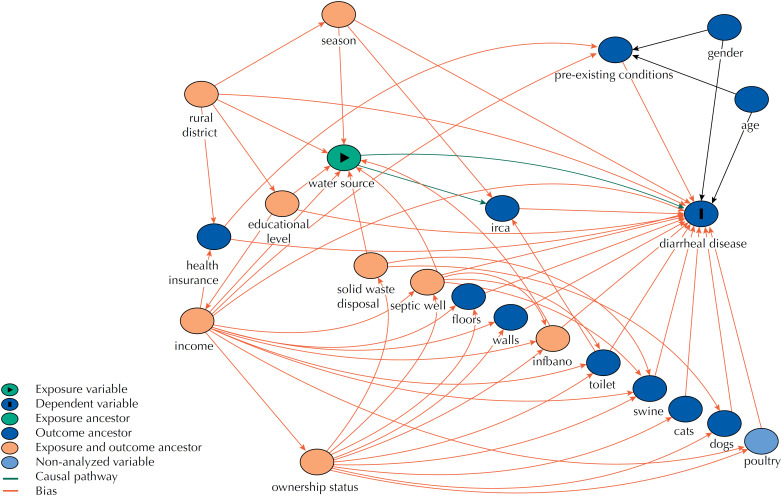
Directed acyclic graph of diarrheal disease within the rural context.

A Directed Acyclic Graph (DAG) was elaborated using the web version of DAGitty software ^20^ when evaluating the association between the diarrheal disease and the exposure variables to identify potential causal mechanisms. In the diagram ( Figure 1 ), conceptually pertinent variables were included based on a literature review and context of the study area. The DAG resulted in 188 testable implications, which were compared against the database, correcting the statistical significance through the Holm-Bonferroni method (reduced from a significance level of 0.05). Not one of the independence implications evaluated was rejected, which suggests consistency between DAG and the dataset ^20^ .

The categorical variables were reported in frequencies and percentages, while the quantitative variable was chosen to be reported with median range and interquartile range, as they have shown a distribution significantly different from normal. Fisher's exact tests were performed to evaluate association between qualitative variables, and Mann-Withney or Kruskall-Wallis tests were performed on quantitative variables.

For each independent variable, a univariable model for diarrheal disease was obtained by using the robust Poisson regression. The measure of association used was the Prevalence Ratio (PR) with a 95% confidence interval (95%CI). To estimate the total effect of each one of the exposure variables, a multivariable robust Poisson regression model was specified adjusting for the variables suggested by DAG. Individuals whose health conditions (diarrheal disease) was unknown were not included in the model. Due to the high prevalence of diarrheal disease in children under five years old and since after this age a clear increase was not observed, the variable age was analyzed as a dichotomous variable.

Although residents were the analysis units, some variables were measured in the household context level. Thus, in the regression models, the option “cluster” was used to account for the household aggregation. A significance level of 0.05 was established.

The resulting data were analyzed in Stata Release 12 (Stata, Corp). This study was approved by the Research Ethics Committee of Los Andes University under the Act no. 965, dated October 30, 2017. An informed consent form was read to the respondents before the survey. Non-disclosure agreements were celebrated by the collaborators of the project. Codes were assigned to the survey forms and samples so that people's identities could be protected.

## RESULTS

Of the 45 censused households, 42 engaged in the study. Altogether 176 people lived in the two rural districts, aged between 0 and 78 years old. Table 1 summarizes the characteristics of the households and of the study population.

**Table 1:** Demographic and household characteristics in the rural districts of El Cascajo and La Delfina, Colombia, 2017–2018

Characteristics		El Cascajo	La Delfina
Number of households		34	8
Household residents		4 ± 2	4 ± 1
Years of residence		6 ± 7	6 ± 8
Rooms per household		2 ± 1	2 ± 0
Property ownership	Yes	24 (70.6)	4 (50.0)
Season	Dry	12 (35.3)	4 (50.0)
Fuel for cooking	Firewood	26 (76.5)	8 (100.0)
	Bottled gas	20 (58.8)	0 (0.0)
Floor	Dirt	27 (79.4)	7 (87.5)
Walls	Wood	27 (79.4)	5 (62.5)
	Cement	4 (11.8)	1 (12.5)
	Clay	3 (8.8)	2 (25.0)
Monthly income	≤ MMW a	28 (82.4)	5 (62.50)
Electricity	No	29 (85.3)	5 (62.5)
	Solar panel	4 (11.8)	3 (37.5)
	Power generator	1 (2.9)	0 (0.0)
Has at least one of the members finished high school?	Yes	14 (41.2)	2 (25.0)
Domestic animals	Poultry	32 (94.1)	8 (100.0)
	Swine	18 (52.9)	6 (75.0)
	Cat	16 (47.1)	5 (62.5)
	Dog	26 (76.5)	7 (87.5)
Waste disposal	Burning	27 (79.4)	6 (75.0)
	Open air	16 (47.1)	3 (37.5)
Toilet facilities	Yes	14 (41.2)	6 (75.0)
Sink	Yes	4 (11.8)	5 (62.5)
Water source b	Rain	9 (26.5)	1 (12.5)
	Artificial pond	10 (29.4)	0 (0.0)
	Deep well	14 (41.2)	0 (0.0)
	River	1 (2.9)	7 (87.5)
Water treatment	Yes	4 (11.8)	0 (0.0)
Water treatment frequency	Always	1 (2.9)	0(0.0)
Water storage	Plastic	24 (70.6)	5 (62.5)
	Metal	2 (5.9)	3 (37.5)
	Clay	6 (17.7)	0 (0.0)
	Cement	2 (5.9)	0 (0.0)
Water issues	Odor	7 (20.6)	4 (50.0)
	Taste	10 (29.4)	5 (62.5)
	Color	15 (44.1)	4 (50.0)
	Other c	2 (5.9)	0 (0.0)
Water quality	IRCA d	60.8 ± 9.1	73.7 ± 5.3
	High risk level	29 (85.3)	8 (100.0)
	Unsanitary	5 (14.7)	0 (0.0)
Number of people		149	27
Age (in years) e		22 ± 15	24 ± 26
	0–4	13 (8.7)	4 (14.8)
	5–12	34 (22.8)	4 (14.8)
	13–26	35 (85.37)	6 (22.2)
	27–59	56 (37.6)	6 (22.2)
	> 60	10 (6.7)	7 (25.9)
Gender	Male	83 (55.7)	17 (63.0)
	Female	66 (44.3)	10 (37.0)
Able to read and write	Yes	121 (81.2)	21 (77.8)
Serviced by a healthcare system	Yes	142 (95.3)	25 (92.6)
Symptomatology (cases)	Diarrhoea f	12 (8.1)	1 (3.7)
	Pre-existing condition	16 (10.7)	2 (7.4)

Median ± Interquartile range, N (%).

a MMW: minimum monthly wage (2018 being the reference year = 238.45 USD; 1 USD = 3,276 COP).

b Water source being used at the moment of the interview, it varies in the households according to the season.

c Presence of animals such as frogs and worms.

d IRCA: Water Quality Risk for Human Consumption Index (IRCA, in the Spanish acronym).

e Without age information for one person.

f Lack of information on diarrhea occurrences for three people.

Out of 105 people over 16 years old, 14.3% had never attended school, 21.9% finished high school and 2.9% started a technical or vocational career. Out of the household chiefs, 78% of men and 86.5% of women were able to read and write.

Dirt was the predominant material on the floors, so as wood was on the walls. Wall materials presented a statistical significance to the income of the household. Forty four percent of the households with incomes higher than the minimum wage had their walls built in cement. Out of those with incomes lower than the minimum wage, 3% had walls in cement.

None of the households of the rural districts have access to natural gas and electricity supplies. All households in La Delfina use firewood for cooking. In El Cascajo, some households presented a combined usage of firewood and bottled gas.

In the totality of households there was at least one domestic animal (poultry, dog, pig, and/or cat), which wandered freely inside and outside of the households.

None of the households had access to a water supply network. Water supply was classified under four types of individual sources: rainwater, artificial pond (open and artificial deposit to retain rainwater), deep well, and river. In four of the households, they treated water occasionally by applying sodium hypochlorite or alum. The source type presented statistical significance to the rural district, being the river water predominant in La Delfina (7/8) and the water from deep wells (14/34) predominant in El Cascajo. Equally, the weather showed a statistical significance to the type of source, with a higher occurrence of the use of artificial pond water (6/16) during the dry season and rainwater (10/26) during rainy season.

**Figure 2:**
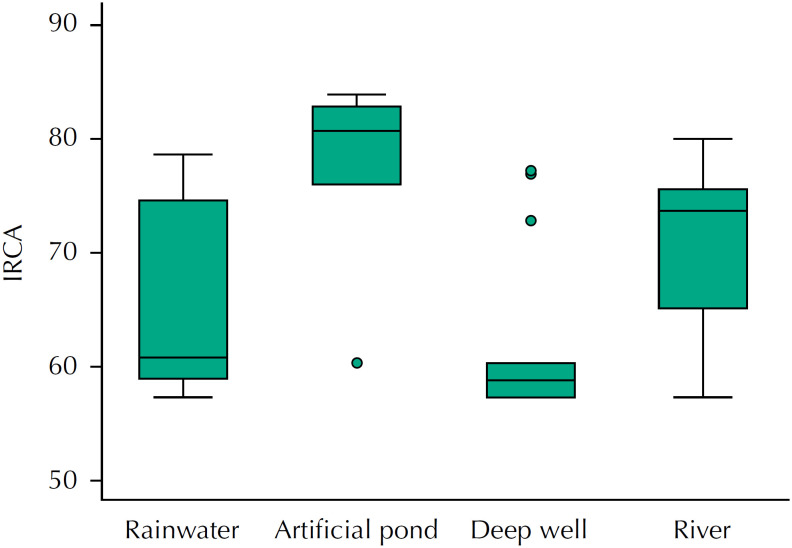
*Índice de Riesgo de la Calidad del Agua para Consumo Humano* (IRCA – Water Quality Risk for Human Consumption Index) from water sources in the rural districts of El Cascajo and La Delfina, Colombia, 2017–2018.

All water samples presented *Escherichia coli* , total coliforms, and aerobic mesophilic. All IRCA results were classified as high risk and unfit for human consumption, being the latter entirely collected from artificial ponds ( Figure 2 ).

Statistical significance difference was found regarding the presence of sinks in the two rural districts, with a higher presence in La Delfina (62.5%) compared with El Cascajo (11.8%). The same was observed regarding the presence of septic tanks, with 87.5% and 44.1%, respectively. The waste was burnt down in most households (78.6%) and some of them employed a combination of burning and open air disposals (28.6%).

During the period, 7.4% cases of diarrheal disease were identified. In addition, 10.2% cases of pre-existing conditions were reported. Data regarding three people were not provided, as they could not answer the questionnaire. The diarrheal disease did not present statistical significance to the cases of pre-existing conditions nor to the rural districts. The prevalence of diarrheal disease was 6.1% in men and 9.5% in women and was unrelated to gender.

In the univariable analysis, the variables season and children under five years old were the only ones whose results showed statistical significance ( Table 2 ).

**Table 2:** Prevalence ratio with no adjustment to diarrheal disease associated with exposure variables in the rural districts of El Cascajo and La Delfina, Colombia, 2017–2018.

Exposure variable		Gastrointestinal diseases				Total (N) a	PR b	95%CI c
		Yes		No				
		N	%	N	%			
Season	Rainy	4	3.5	109	96.5	113	0.24	0.07–0.84 *
	Dry (Ref)	9	15.0	51	85.0	60	1.00	NA
Rural district	La Delfina	1	3.7	26	96.3	27	0.45	0.05–3.98
	El Cascajo (Ref)	12	8.2	134	91.8	146	1.00	NA
Property ownership status	Owner	8	7.5	99	92.5	107	0.99	0.24–4.09
	Others (Ref)	5	7.6	61	92.4	66	1.00	NA
Floor	Cement	1	2.2	45	97.8	46	0.23	0.03–1.65
	Dirt (Ref)	12	9.5	115	90.6	127	1.00	NA
Walls	Cement	1	5.0	19	95.0	20	0.64	0.10–4.02
	Wood/Clay (Ref)	12	7.8	141	92.2	153	1.00	NA
Educational level d	Yes	4	6.4	59	93.7	63	0.78	0.22–2.78
	No (Ref)	9	8.2	101	91.8	110	1.00	NA
Income	> than one MMW	2	4.6	42	95.5	44	0.54	0.13–2.22
	≤ than one MMW (Ref)	11	8.5	118	91.5	129	1.00	NA
Swine	Yes	4	3.8	102	96.2	106	0.28	0.08–1.02
	No (Ref)	9	13.4	58	86.6	67	1.00	NA
Dog	Yes	7	5.3	124	94.7	131	0.37	0.09–1.58
	No (Ref)	6	14.3	36	85.7	42	1.00	NA
Cat	Yes	8	9.8	74	90.2	82	1.78	0.33–9.62
	No (Ref)	5	5.5	86	94.5	91	1.00	NA
Gender	Female	7	9.5	67	90.5	74	1.56	0.51–4.75
	Male (Ref)	6	6.1	93	93.9	99	1.00	NA
Age	< 5 years old	4	23.5	13	76.5	17	4.05	1.70–9.68 *
	≥ 5 years old (Ref)	9	5.8	146	94.2	155	1.00	NA
Pre-existing conditions	Yes	2	11.1	16	88.9	18	1.57	0.31–7.89
	No (Ref)	11	7.10	144	92.9	155	1.00	NA
Health insurance	Subscriber	12	7.3	152	92.7	164	0.65	0.09–4.64
	No (Ref)	1	11.1	8	88.9	9	1.00	NA
Water source	Rain	1	2.3	42	97.7	43	0.74	0.05–11.49
	Artificial pond	5	12.5	35	87.5	40	4	0.33–49.04
	Deep well	6	10.3	52	89.7	58	3.31	0.37–29.47
	River (Ref)	1	3.1	31	96.9	32	1.00	NA
Solid waste disposal	Burning	5	5.7	83	94.3	88	1.12	0.27–4.60
	Open air	5	19.2	21	80.8	26	3.78	0.74–19.37
	Both (Ref)	3	5.1	56	94.9	59	1.00	NA
Sinks	Yes	1	3.6	27	96.4	28	0.43	0.05–3.60
	No (Ref)	12	8.3	133	91.7	145	1.00	NA
Toilet facilities	Yes	3	3.5	82	96.5	85	0.31	0.08–1.24
	No (Ref)	10	11.4	78	88.6	88	1.00	NA
Septic tank	Yes	4	5.0	76	95.0	80	0.52	0.14–1.93
	No (Ref)	9	9.7	84	90.3	93	1.00	NA
Risk level	Unsanitary	4	18.2	18	81.8	22	3.05	0.51–18.2
	High (Ref)	9	6.0	142	94.0	151	1.00	NA

NA: not applicable; Ref: reference category

a Lack of information on diarrheal disease on three people. Estimates based on a total of 103 people.

b PR: prevalence ratio

c 95%CI: 95%confidence interval.

d Educational level: at least one resident graduated from high school in the household.

* Statistical significance at α = 0,05.

When ajusted by covariates, the variables rainy season, presence of swine, presence of toilet facilities, children under five years old, water from a tank source, and well water presented statistical significance to diarrheal disease ( Table 3 ).

**Table 3:** Prevalence ratio adjusted to diarrheal disease associated with exposure variables in the rural districts of El Cascajo and La Delfina, Colombia, 2017–2018.

Exposure variable		PR a	95%CI b	Set of variables c
Season	Rainy	0.24	0.07–0.85 *	Rural district
Rural district	La Delfina	0.45	0.05–3.98	NA
Ownership status	Owner	0.95	0.23–3.86	Income
Floors	Cement	0.20	0.03–1.32	Income, ownership status
Walls	Cement	0.96	0.14–6.83	Income, ownership status
Educational level	One person graduated from high school in the household	0.74	0.21–2.64	Rural District
Income	Over than a monthly minimum wage	0.54	0.13–2.22	Educational level
Swine	Yes	0.20	0.05–0.74 *	Season, toilet, income, educational level, dogs, presence of septic tank, water source, ownership status, rural district
Dogs	Yes	0.35	0.10–1.26	Swine, season, toilet facilities, income, educational level, presence of septic tank, ownership status, water source
Cat	Yes	1.84	0.40–8.46	Ownership status
Gender	Female	1.56	0.51–4.75	NA
Age	< 5 years old	4.05	1.70–9.68 *	NA
Pre-existing conditions	Yes	2.21	0.34–14.47	Age, gender, income, health insurance
Health insurance	Yes	0.59	0.08–4.45	Income, rural district
Water source	Rainwater	5.20	0,60-45,18	Swine, season, toilet facilities, income, educational level, dog, presence of septic tank, ownership status, rural district
	Artificial pond	11.47	1,27-103,29 *	
	Deep well	16.90	2,45-116,67 *	
	River	1.00	NA	
Solid waste disposal	Burning	1.14	0.26–4.95	Ownership status
	Open air	3.97	0.74–21.35	
	Both	1.00	NA	
Sinks	Yes	0.60	0.06–6.10	Income, ownership status, toilet facilities
Toilet	Yes	0.23	0.06–0.96 *	Income, presence of septic tank, ownership status
Septic tank	Yes	0.55	0.15–1.99	Income, ownership status
Risk level	Unsanitary	1.13	0.13–9.43	Season, presence of sinks, water source

a PR: prevalence ratio

b 95%CI: 95% confidence interval.

c Variable set for adjusting results of DAG

* Statistical significance at α = 0.05.

## DISCUSSION

Poor basic sanitation and water supply conditions for human consumption were observed in the rural districts located in the rural Caribbean region of Colombia. The households had no waste collection services, sewage and water supply systems nor a community service of water supply. Individual water supply systems were used, from different sources (rainwater, deep well, artificial pond and/or river), in some cases, located hundreds of feet away. This makes transportation harder and limits the amount of water supplied. Such characteristics match those of the peri-urban and rural populations in developing countries, as reported by Razzolini y Günther ^21^ .

Water samples collected in several sources of supply had presented different qualities, influenced by sources and contamination spots. A high indicator of fecal coliforms was a universal factor in the water samplings. Such contamination is commonly found in rural areas due to cattle breeding ^22^ , open defecation ^23^ , personal hygiene habits carried out close to or inside the water source and improper manipulation, transportation and storage of water from source to households ^24^^,^^25^ .

The prevalence of diarrheal disease in the rural districts of El Cascajo and La Delfina was 7.5% at all ages. Regardless, in children under five years old, this figure went as higher as 23.5%, which is 91% higher than the medium prevalence rate reported in Colombia (12.3%) ^26^ , yet similar to the prevalence rates reported in low-income countries such as Burundi, Cameroon, Haiti, Nigeria, Uganda, and Senegal ^26^ .

We also found a larger occurrence of diarrheal disease during the dry season in comparison to the rainy season, a finding that matches the evidence reported by Wilopo et al. ^27^ and Vila et al. ^28^ . Such association can be explained through the increase in usage of artificial pond water as a water supply during this season. The same source is used as water trough for the animals, which transfer microbial load into the water.

Divergently, some studies had detected an increased frequency of diarrheal disease during the rainy season ^25^^,^^29^ , which can be explained for the dragging of the surrounding pollution caused by the drainage of the water. However, in this study, the reduction of diarrheal cases during the rainy season may be influenced by preference of consuming rainwater rather than water affected by drainage (rivers and artificial ponds).

In the non-adjusted model, none of the water sources presented statistical significance to the cases of diarrheal disease. In the adjusted model, we observed statistical significance in the water sourced from artificial ponds and deep wells, with a prevalence ratio that suggests them to be risk factors. The previous pattern reflected an error that could be mitigated by the variables set used in the model adjustment, including the variables season and rural district. The first variable could influence the exposure to the diarrheal disease by favoring the consumption of rainwater during the wet season. Moreover, the rural district may influence the access to certain types of water sources according to its geography.

The possession of toilet facilities was associated with the outcome, seeming to be a protective factor. Even though further basic sanitation variables had not presented statistical significance, we observed a minor prevalence in the households that had sinks and/or septic tanks installed.

The keeping of swine turned out to be a protective factor in the adjusted model. Some studies have found domestic animals to be a risk factor ^6^ . However, we consider that the keeping of swine could be an indicator of better socioeconomic status within the surveyed families and, perhaps, of better hygiene conditions and habits.

Future studies to analyze the association between diarrheal disease and the keeping of swine can diverge from our findings and determinate whether the presence of swine is a protective factor from diarrheal disease in places with poor basic sanitation conditions.

We recorded high IRCA values in water sources for human consumption. This would explain the strong associations found between some of the water sources and the frequency of diarrheal disease. Thus, our results show the impacts of low rates in water treatment, reflecting the limited knowledge available on the health risks of such exposures.

These results should promote local and regional programs in the long run to improve the water quality at consumption points, with actions such as solar water disinfection, coagulation, sodium chloride treatment or boiling. Such interventions must be paired with educational campaigns about hygiene and basic sanitation aimed at: a) advising the community about the health risks of consuming poor quality water and poor basic sanitation conditions; b) spreading knowledge on proper manipulation, transportation and storage of water, and c) raising awareness about the responsible keeping of domestic animals.

These actions must be transitory measures towards stronger solutions that generate differential and cross-sectional policies for the rural sector, which take into full account the water supply and quality issues along with the proper disposal of waste and garbage. In order to assure the integration of the different sectors, such policies may incorporate strategies such as: a) incentive for the creation of social business focused on the design of alternative technologies rather than conventional solutions; b) educational programs in rural schools about hygiene and basic sanitation; c) technical assistance to the community; d) engagement of the community so that it plays an active role in the report of the water quality control and cases of the diarrheal disease. All of these policies aim to improve water quality for consumption, controlling thus the diarrheal disease, which hits especially the pediatric community.

This study has a few limitations. Even though the seven-day recall window is commonly used in the practice to prevent the under-record of information ^30^ , said period may be too short to get representative information about the incidence of the diarrheal disease in the families. Under-records of the diarrheal disease also could have been presented by the person answering the questionnaire, due to the unawareness of symptomatology presented by other individuals or by the shame of revealing such information.

In addition, when the physical, chemical, and microbiological properties of the water in each household was measured, the resulting water quality does not represent the water quality consumed in the seven days before the survey. However, the IRCA estimated shows the high risk posed by the water for human consumption in both rural districts at the moment of the study.

Moreover, the limited size of the sampling had reflected a limited precision and, probably, a limited power in identifying further factors associated with the diarrheal disease. Nevertheless, the outcome exposes a diagnosis of the study area. Likewise, the directed acyclic graph elaborated can turn into a useful conceptual tool for future research that share the same context.

The long distances between households and the difficult transport routes that characterize the rural area not only restrict the implementation of water supply networks and basic sanitation, but also hinder the logistics for these types of studies. These results are expected to be a referential evidence and to make easier for decision makers and other researchers to carry out complementary studies on the matter.

Despite the efforts deployed on the issue of drinking water and improvement of living conditions of the most vulnerable population of Colombia, poor conditions regarding water supply, hygiene and basic sanitation are still seen in the rural area. There is an urge for effective design of policies to contribute to the improvement of environmental and sanitation conditions in households and reduce the prevalence of diarrheal disease, especially in children under five years old.
