# Supplementary material for: Factors associated with diarrheal disease in the rural Caribbean region of Colombia
Source: Rev Saude Publica. 2020 Sep 24;54:90. doi: 10.11606/s1518-8787.2020054002054 (PMC7524207; doi:10.11606/s1518-8787.2020054002054)
Supplement: Translation

# Translation: Factores asociados con enfermedad diarreica en área rural del Caribe colombiano

**Authors:** Maria-Angelica Galezzo, Wanda Maria Risso Günther, Fredi Alexander Diaz-Quijano, Manuel Rodriguez Susa

**Affiliations:** I BogotáDC Colombia Universidad de los Andes. Centro de Investigaciones en Ingeniería Ambiental. Departamento de Ingeniería Civil y Ambiental. Bogotá, DC, Colombia; II São PauloSP Brasil Universidade de São Paulo. Laboratório de Gestão Ambiental, Inovação e Sustentabilidade. São Paulo, SP, Brasil; III São PauloSP Brasil Universidade de São Paulo. Faculdade de Saúde Pública. Laboratório de Inferência Causal em Epidemiologia. São Paulo, SP, Brasil

**Keywords:** Diarrea Infantil, epidemiología, Diarrea, epidemiología, Factores de Riesgo, Factores Socioeconómicos, Saneamiento Rural, Salud Rural

## Abstract

**OBJETIVO:**

Analizar factores asociados con enfermedad diarreica en área rural del Caribe colombiano.

**MÉTODO:**

Estudio transversal en área rural dispersa del departamento del Cesar, Colombia, entre noviembre de 2017 y junio de 2018. Se indagó sobre morbilidad auto-reportada de enfermedad diarreica y se recolectaron y analizaron muestras de agua en 42 domicilios. Fue realizado un análisis descriptivo de condiciones socioeconómicas, ambientales y sanitarias y evaluamos su asociación con enfermedad diarreica mediante modelo robusto de regresión de Poisson. Cada modelo fue ajustado con variables sugeridas por diagramas causales específicos.

**RESULTADOS:**

Se evidenciaron condiciones precarias de abastecimiento de agua, higiene y saneamiento básico en la zona de estudio. Todas las muestras de agua se clasificaron entre los niveles de riesgo alto e inviable sanitariamente. La prevalencia de enfermedad diarreica fue 7,5% en todas las edades y 23,5% en niños menores de cinco años. Las variables estación lluviosa (RP = 0,24; IC95% 0,07–0,85), niños menores de cinco años (RP = 4,05; IC95% 1,70–9,68), abastecimiento de agua desde pozo profundo (RP = 16,90; IC95% 2,45–116,67), abastecimiento de agua desde estanco (RP = 11,47; IC95% 1,27–103,29), tenencia de baño (RPA = 0,23; IC95% 0,06–0,96) y presencia de cerdos (RP = 0,20; IC95% 0,05–0,74) mostraron asociaciones estadísticamente significativas con la ocurrencia de enfermedad diarreica.

**CONCLUSIÓN:**

Condiciones de abastecimiento de agua, higiene y saneamiento básico estuvieron asociadas con la ocurrencia de enfermedad diarreica, afectando alrededor de un cuarto de la población menor de cinco años. Urge un diseño efectivo de políticas que contribuyan al mejoramiento de condiciones ambientales y saneamiento en áreas rurales.

## INTRODUCCIÓN

Bajo nivel socioeconómico ^1^^,^^2^ , deficiente saneamiento básico ^2^^-^^4^ , consumo de agua insegura ^3^^,^^4^ , hábitos de higiene inadecuados ^2^^-^^5^ , tenencia de animales domésticos ^6^ , entre otros factores, hacen del área rural latinoamericana, asiática y africana una zona propicia para el desarrollo de enfermedades transmitidas por el agua. La enfermedad diarreica se destaca con aproximadamente 525.000 casos de mortalidad y 1.700 millones de morbilidad en niños menores de cinco años alrededor del mundo ^7^ .

Las comunidades rurales representan el 83,0% de la población mundial que carece de acceso a una fuente de agua mejorada, y el 71,0% de la que no cuenta con saneamiento ^8^ . El 57,0% de las viviendas ubicadas en el área rural dispersa en Colombia carecen de acceso a agua potable y el 94,0%, de sistema de alcantarillado; porcentajes que ascienden a 87,0% y 98,0%, respectivamente, en el departamento del Cesar ^9^ .

En el marco del cumplimiento de la agenda de Objetivos de Desarrollo del Milenio (finalizada en 2015), en Colombia se crearon e implementaron proyectos como: Red Unidos para la reducción de la pobreza, Sistema para la Protección y Control de la Calidad del Agua para consumo humano y Sistema Nacional de Vigilancia en Salud Pública, entre otros. Estos proyectos contribuyen a mejorar condiciones de vida de la población. Sin embargo, se requiere de mayores esfuerzos en la elaboración de políticas diferenciales e intersectoriales que se ajusten al contexto de la ruralidad y contribuyan a la reducción de la brecha actual ^10^^,^^11^ , promoviendo mayor equidad y mejoras en la salud de la población rural.

Para estos fines, es esencial reconocer determinantes de la enfermedad diarreica en zonas rurales para la proposición de soluciones apropiadas y de mayor alcance sanitario y ambiental. Atendiendo a esta necesidad, el objetivo de este estudio fue analizar factores asociados con enfermedad diarreica en área rural del Caribe colombiano.

## MÉTODOS

Este estudio fue desarrollado en las veredas La Delfina (latitud de 10º 19' N, longitud de 73º 28' W) y El Cascajo (latitud de 9° 25' N, longitud de 73° 57' W), localizadas a 40 km y 194 km, respectivamente, de la ciudad de Valledupar, capital del departamento del Cesar. La selección de la zona de estudio se realizó priorizando dos municipios por medio de los métodos Electre ^12^ y Topsis ^12^ . Los criterios de selección fueron: i) menor cobertura de acueducto, ii) mayor número de habitantes, iii) mayor tasa de mortalidad infantil (1998–2015) y iv) menor Índice de Pobreza Multidimensional (IPM); los cuales fueron previamente ponderados por medio de los métodos CRITIC ^13^ y Entropía ^14^ . Los datos analizados correspondieron a la zona rural de cada municipio.

Posteriormente, seleccionamos dos veredas (menor unidad administrativa) por medio de un Proceso Analítico Jerárquico ^12^ , en donde involucramos actores conocedores del área rural de los municipios priorizados. Los criterios valorados fueron i) menor cobertura de acueducto, ii) mayor escasez de agua, iii) fácil accesibilidad y iv) mayor participación comunitaria.

Las veredas fueron censadas en septiembre de 2017 para identificar el tamaño de la población, condiciones sanitarias y fuente de suministro de agua para consumo humano. Un sistema de información geográfico (SIG) fue elaborado con las coordenadas de las viviendas. Los resultados del censo fueron utilizados para elaborar el instrumento de recolección de datos e identificar el tipo de muestreo para la colecta de agua.

Un cuestionario se elaboró tomando como base el formulario aplicado en el Censo Nacional Agropecuario (CNA) de 2014 ^15^ y otros estudios similares ^16^^,^^17^ , que fueron adaptados a la zona de estudio. El cuestionario comprendió 68 preguntas relacionadas con: aspectos demográficos de la población; condiciones socio económicas, sanitarias y de suministro y almacenamiento de agua en las viviendas. Se realizó una prueba piloto previa al proceso de entrevistas y se realizaron ajustes requeridos.

Para abarcar estación seca y lluviosa, fueron analizados valores mensuales de precipitación (1985–2015) de estaciones meteorológicas cercanas a la zona de estudio. Las entrevistas y recolección de muestras fueron realizadas aleatoriamente en noviembre de 2017, y marzo y junio de 2018.

La entrevista fue realizada por un equipo entrenado (un estudiante doctoral y tres estudiantes graduados en ingeniería ambiental) al responsable del hogar o a una persona residente mayor de 18 años. Las muestras de agua fueron colectadas desde recipientes de almacenamiento en cada hogar bajo lineamientos de normatividad colombiana ^18^ . Los análisis de parámetros fisicoquímicos y microbiológicos fueron realizados en el laboratorio de Ingeniería Ambiental de la Universidad de los Andes.

Además de las condiciones de las viviendas, se indagó acerca de la morbilidad relacionada con casos de diarrea, ocurridos en los siete días previos a la visita. Se preguntó acerca de condiciones médicas preexistentes para evitar interferencias en el momento de identificar los casos de enfermedad diarreica: embarazo, cirugía en el estómago, úlceras estomacales o alguna enfermedad relacionada con el colón.

Para resumir la calidad del agua, calculamos el Índice de Riesgo de Calidad del Agua para consumo humano (IRCA), siguiendo los lineamientos de Resolución 2115 de 2007 ^19^ . Los valores del IRCA oscilan entre 0 a 100 y se clasifican según el nivel de riesgo que puede ocasionar el consumo de agua en la salud de las personas: sin riesgo (≤ 5), riesgo bajo (5,1 a 14), riesgo medio (14,1 a 35), riesgo alto (35,1 a 80) e inviable sanitariamente (80,1 a 100).

Enfermedad diarreica fue la variable dependiente, la cual se definió como la ocurrencia de tres o más defecaciones blandas en un periodo de 24 horas. La prevalencia de periodo de enfermedad diarreica en los siete días previos a la recolección de datos fue calculada como la proporción de habitantes que presentaron al menos un evento en el periodo de los siete días previos a la entrevista.

Las condiciones socioeconómicas, de saneamiento básico y de abastecimiento de agua en la vivienda, junto con las condiciones demográficas recolectadas, fueron variables de exposición.

La ausencia de aves en las viviendas descartó perfectamente la ocurrencia del evento, dado que todos los casos de enfermedad diarreica se presentaron en viviendas con presencia de aves. Por lo tanto, esta variable no se incluyó en el modelo debido a la imposibilidad de obtener medidas de asociación ajustadas.

Se elaboró un Gráfico Acíclico Dirigido (DAG: *Directed Acyclic Graph* ) en la versión web del software DAGitty ^20^ al evaluar la asociación entre enfermedad diarreica y las variables de exposición para identificar los potenciales mecanismos de confusión. Se incluyeron variables conceptualmente pertinentes con base en una revisión bibliográfica y el contexto de la zona de estudio en este diagrama ( Figura 1 ). Resultaron del DAG 188 implicaciones evaluables, las cuales fueron contrastadas con la base de datos, corrigiendo el nivel de significancia por medio del método Holm-Bonferroni (reducido desde un nivel de 0,05). Ninguna de las independencias evaluadas fue rechazada, sugiriendo consistencia entre el DAG y el conjunto de datos ^20^ .

**Figura 1:** Gráfico acíclico dirigido de enfermedad diarreica en contexto rural.

Las variables categóricas se reportaron en frecuencias y porcentajes; mientras que para las cuantitativas se prefirió reportar la mediana y el rango intercuartil, debido a que revelaron una distribución significativamente diferente a la normal. Pruebas de Fisher fueron aplicadas para evaluar asociación entre variables cualitativas; así como pruebas de *Mann-Withney* o *Kruskal Wallis* para variables cuantitativas.

Para cada variable independiente, se obtuvo un modelo univariable robusto de regresión de Poisson con relación a enfermedad diarreica. La medida de asociación utilizada fue la Razón de Prevalencia (RP) con un intervalo de 95% de confianza (IC95%). Para estimar el efecto total de cada una de las variables de exposición, se obtuvo un modelo multivariable robusto de regresión de Poisson ajustado con las variables sugeridas por el DAG. Los individuos cuya condición de salud (enfermedad diarreica) fue desconocida no fueron incluidos dentro del modelo. Debido a la alta prevalencia de enfermedad diarreica en menores de cinco años, y a que después de esa edad no se observó un claro gradiente, la variable edad fue analizada como dicotómica.

Aunque las unidades de análisis fueron los habitantes, algunas variables fueron medidas en un nivel contextual de vivienda. Por lo anterior, en los modelos de regresión se utilizó la opción “cluster” para considerar la agregación por vivienda. Fue preestablecido un nivel de significancia de 0,05.

Los datos obtenidos fueron analizados en *Stata* versión 12 ( *Stata, Corp* ). Este estudio fue aprobado por el Comité de Ética de la Investigación de la Universidad de los Andes por medio de Acta Nº 965, del 30 de octubre de 2017. Un consentimiento informado fue leído a las personas encuestadas antes de aplicar el cuestionario. Actas de confidencialidad fueron diligenciadas por los colaboradores del proyecto. A los cuestionarios y formatos de toma de muestra les fueron asignados códigos para proteger la identidad de las personas.

## RESULTADOS

De las 45 viviendas que fueron censadas, 42 participaron en el estudio. Un total de 176 personas habitaban las dos veredas, en edades entre cero y 78 años. La tabla 1 resume las características de las viviendas y de la población estudiada.

**Tabla 1:** Características demográficas y de viviendas en las veredas El Cascajo y La Delfina, Colombia, 2017–2018.

Características		El Cascajo	La Delfina
Número de viviendas		34	8
Habitantes por vivienda		4 ± 2	4 ± 1
Años de residencia		6 ± 7	6 ± 8
Habitaciones por vivienda		2 ± 1	2 ± 0
Casa propia	Sí	24 (70,6)	4 (50,0)
Estación	Seca	12 (35,3)	4 (50,0)
Combustible para cocinar	Leña	26 (76,5)	8 (100,0)
	Gas embotellado	20 (58,8)	0 (0,0)
Piso	Tierra	27 (79,4)	7 (87,5)
Pared	Madera	27 (79,4)	5 (62,5)
	Cemento	4 (11,8)	1 (12,5)
	Barro	3 (8,8)	2 (25,0)
Ingresos mensuales	≤ SMML a	28 (82,4)	5 (62,50)
Energía eléctrica	No	29 (85,3)	5 (62,5)
	Panel solar	4 (11,8)	3 (37,5)
	Planta eléctrica	1 (2,9)	0 (0,0)
Por lo menos una persona terminó la secundaria	Sí	14 (41,2)	2 (25,0)
Animales domésticos	Aves de corral	32 (94,1)	8 (100,0)
	Cerdo	18 (52,9)	6 (75,0)
	Gato	16 (47,1)	5 (62,5)
	Perro	26 (76,5)	7 (87,5)
Disposición de residuos	Quemados	27 (79,4)	6 (75,0)
	Cielo abierto	16 (47,1)	3 (37,5)
Baño	Sí	14 (41,2)	6 (75,0)
Lavamanos	Sí	4 (11,8)	5 (62,5)
Fuente de agua b	Lluvia	9 (26,5)	1 (12,5)
	Estanco	10 (29,4)	0 (0,0)
	Pozo	14 (41,2)	0 (0,0)
	Río	1 (2,9)	7 (87,5)
Tratamiento de agua	Sí	4 (11,8)	0 (0,0)
Frecuencia de tratamiento	Siempre	1 (2,9)	0(0,0)
Almacenamiento de agua	Plástico	24 (70,6)	5 (62,5)
	Metal	2 (5,9)	3 (37,5)
	Arcilla	6 (17,7)	0 (0,0)
	Cemento	2 (5,9)	0 (0,0)
Problemas con el agua	Olor	7 (20,6)	4 (50,0)
	Sabor	10 (29,4)	5 (62,5)
	Color	15 (44,1)	4 (50,0)
	Otro c	2 (5,9)	0 (0,0)
Calidad del agua	IRCA d	60,8 ± 9,1	73,7 ± 5,3
	Nivel de riesgo alto	29 (85,3)	8 (100,0)
	Inviable sanitariamente	5 (14,7)	0 (0,0)
Número de personas		149	27
Edad (años) e		22 ± 15	24 ± 26
	0–4	13 (8,7)	4 (14,8)
	5–12	34 (22,8)	4 (14,8)
	13–26	35 (85,37)	6 (22,2)
	27–59	56 (37,6)	6 (22,2)
	> 60	10 (6,7)	7 (25,9)
Sexo	Masculino	83 (55,7)	17 (63,0)
	Femenino	66 (44,3)	10 (37,0)
Sabe leer y escribir	Sí	121 (81,2)	21 (77,8)
Afiliación a sistema de salud	Sí	142 (95,3)	25 (92,6)
Sintomatología (casos)	Diarrea f	12 (8,1)	1 (3,7)
	Condición médica preexistente	16 (10,7)	2 (7,4)

Mediana ± Rango Intercuartil, N (%).

a SMML: salario mínimo mensual legal (2018 como año de referencia = USD 238,45; 1 USD = 3.276).

b Fuente utilizada en el momento de la entrevista, varía en las viviendas de acuerdo con la estación.

c Presencia de animales como ranas y gusanos.

d IRCA: Índice del Riesgo de la Calidad de Agua para consumo humano.

e Una persona sin información de edad.

f Tres personas sin información de ocurrencia de diarrea.

De las 105 personas mayores de 16 años: 14,3% nunca fueron a la escuela, 21,9% terminaron la secundaria y 2,9% empezaron una carrera técnica o tecnológica. Entre las personas responsables del hogar, 78,0% de los hombres y 86,5% de las mujeres sabían leer y escribir.

La tierra fue el material predominante de los pisos y la madera el de las paredes. El tipo de pared presentó asociación estadísticamente significativa con los ingresos del hogar. El 44,0% de las viviendas cuyos ingresos eran superiores a un salario mínimo tenían construida sus paredes en cemento. En aquellas que obtenían ganancias menores a un salario mínimo, el 3,0% tenían paredes en este material.

En ninguna de las veredas las casas tenían suministro de gas natural y energía eléctrica. Todas las viviendas en La Delfina utilizaban leña para la cocción de alimentos. Por su parte, algunas viviendas combinaban el uso de leña con gas embotellado en El Cascajo.

En todas las viviendas se observó por lo menos un animal doméstico (ave de corral, perro, cerdo y/o gato), los cuales paseaban libremente dentro de las casas y en el exterior de estas.

Ninguna de las casas contaba con red de distribución de agua. El abastecimiento de agua fue provisto desde cuatro tipos de fuentes individuales: agua lluvia, estanco (depósito abierto y artificial que retiene el agua lluvia), pozo profundo y río. En cuatro de las viviendas, manifestaron que realizaban tratamiento de agua esporádicamente por medio de la aplicación de hipoclorito de sodio o alumbre. El tipo de fuente presentó asociación estadísticamente significativa con la vereda, predominando el agua de río en la vereda La Delfina (7/8) y el agua de pozo profundo (14/34) en El Cascajo. De igual manera, la estación climática mostró asociación estadísticamente significativa con el tipo de fuente, con una mayor frecuencia en el uso de agua de estanco (6/16) en época seca y de agua lluvia (10/26) en época lluviosa.

Todas las muestras de agua tuvieron presencia de *Escherichia coli* , coliformes totales y aerobios mesófilos. Todos los valores de IRCA se clasificaron como alto riesgo e inviable sanitariamente, estos últimos todos provenientes de estanco ( Figura 2 ).

**Figura 2:** Índice de Riesgo de la Calidad del Agua (IRCA) de fuentes de abastecimiento en las veredas El Cascajo y La Delfina, Colombia, 2017–2018.

Se encontró diferencia estadísticamente significativa entre tenencia de lavamanos y las dos veredas, con un mayor porcentaje en La Delfina (62,5%) en comparación a El Cascajo (11,8%). El mismo comportamiento fue observado para tenencia de pozo séptico, con respectivamente 87,5% y 44,1%. Los residuos eran quemados en la mayoría de las viviendas (78,6%) y algunas combinaban la quema con la disposición a cielo abierto (28,6%).

Se identificaron 7,4% de casos de enfermedad diarreica para el periodo. Además, se reportaron 10,2% de casos de otras condiciones médicas preexistentes. No fue suministrada información de tres personas por desconocimiento de la persona encuestada. La enfermedad diarreica no presentó asociación estadísticamente significativa con los casos de condiciones preexistentes y tampoco con las veredas. La prevalencia de enfermedad diarreica en los hombres fue de 6,1% y en las mujeres de 9,5% y no estuvo asociada al sexo.

En el análisis univariable (sin ajustar), las variables estación y niños menores de cinco años fueron las únicas cuyos resultados tuvieron asociación estadísticamente significativa ( Tabla 2 ).

**Tabla 2:** Razón de prevalencia sin ajustar de enfermedad diarreica asociadas a variables de exposición en las veredas El Cascajo y La Delfina, Colombia, 2017–2018.

Variable de exposición		Enfermedad gastrointestinal				Total (N) a	RP b	IC95% c
		Sí		No				
		N	%	N	%			
Estación	Lluvia	4	3,5	109	96,5	113	0,24	0,07–0,84 *
	Seca (Ref)	9	15,0	51	85,0	60	1,00	NA
Vereda	Las Delfinas	1	3,7	26	96,3	27	0,45	0,05–3,98
	El Cascajo (Ref)	12	8,2	134	91,8	146	1,00	NA
Tipo de propiedad	Dueño	8	7,5	99	92,5	107	0,99	0,24–4,09
	Otro (Ref)	5	7,6	61	92,4	66	1,00	NA
Tipo de piso	Cemento	1	2,2	45	97,8	46	0,23	0,03–1,65
	Tierra (Ref)	12	9,5	115	90,6	127	1,00	NA
Tipo de pared	Cemento	1	5,0	19	95,0	20	0,64	0,10–4,02
	Madera/Barro (Ref)	12	7,8	141	92,2	153	1,00	NA
Nivel educativo d	Sí	4	6,4	59	93,7	63	0,78	0,22–2,78
	No (Ref)	9	8,2	101	91,8	110	1,00	NA
Ingresos	> a un SMML	2	4,6	42	95,5	44	0,54	0,13–2,22
	≤ a un SMML (Ref)	11	8,5	118	91,5	129	1,00	NA
Cerdo	Sí	4	3,8	102	96,2	106	0,28	0,08–1,02
	No (Ref)	9	13,4	58	86,6	67	1,00	NA
Perro	Sí	7	5,3	124	94,7	131	0,37	0,09–1,58
	No (Ref)	6	14,3	36	85,7	42	1,00	NA
Gato	Sí	8	9,8	74	90,2	82	1,78	0,33–9,62
	No (Ref)	5	5,5	86	94,5	91	1,00	NA
Sexo	Femenino	7	9,5	67	90,5	74	1,56	0,51–4,75
	Masculino (Ref)	6	6,1	93	93,9	99	1,00	NA
Edad	< 5 años	4	23,5	13	76,5	17	4,05	1,70–9,68 *
	≥ 5 años (Ref)	9	5,8	146	94,2	155	1,00	NA
Condiciones preexistentes	Sí	2	11,1	16	88,9	18	1,57	0,31–7,89
	No (Ref)	11	7,10	144	92,9	155	1,00	NA
Seguro de salud	Afiliado	12	7,3	152	92,7	164	0,65	0,09–4,64
	No (Ref)	1	11,1	8	88,9	9	1,00	NA
Tipo de fuente	Lluvia	1	2,3	42	97,7	43	0,74	0,05–11,49
	Estanco	5	12,5	35	87,5	40	4	0,33–49,04
	Pozo profundo	6	10,3	52	89,7	58	3,31	0,37–29,47
	Río (Ref)	1	3,1	31	96,9	32	1,00	NA
Disposición de residuos sólidos	Quemados	5	5,7	83	94,3	88	1,12	0,27–4,60
	Aire libre	5	19,2	21	80,8	26	3,78	0,74–19,37
	Ambos (Ref)	3	5,1	56	94,9	59	1,00	NA
Lavamanos	Sí	1	3,6	27	96,4	28	0,43	0,05–3,60
	No (Ref)	12	8,3	133	91,7	145	1,00	NA
Baño	Sí	3	3,5	82	96,5	85	0,31	0,08–1,24
	No (Ref)	10	11,4	78	88,6	88	1,00	NA
Pozo séptico	Sí	4	5.0	76	95.0	80	0,52	0,14–1,93
	No (Ref)	9	9,7	84	90,3	93	1,00	NA
Nivel de riesgo	Inviable sanitariamente	4	18,2	18	81,8	22	3,05	0,51–18,2
	Alto (Ref)	9	6.0	142	94.0	151	1,00	NA

NA: no aplica; Ref: categoría de referencia

a Tres personas sin información de enfermedad diarreica. Los cálculos se realizan para un total de 103 personas.

b RP: razón de prevalencia.

c IC95%: intervalo de confianza del 95%.

d Nivel educativo: por lo menos una persona terminó secundaria en la vivienda.

* Significancia estadística para α = 0,05.

Cuando se ajustaron las estimaciones, las variables estación lluviosa, presencia de cerdo, tenencia de baño, niños menores de cinco años, abastecimiento de agua desde estanco y abastecimiento de agua desde pozo profundo tuvieron asociación estadísticamente significativa con enfermedad diarreica ( Tabla 3 ).

**Tabla 3:** Razón de prevalencia ajustada de enfermedad diarreica asociadas a variables de exposición en las veredas El Cascajo y La Delfina, Colombia, 2017–2018.

Variable de exposición		RP a	IC95% b	Conjunto de variables c
Estación	Lluvia	0,24	0,07–0,85 *	Vereda
Vereda	La Delfina	0,45	0,05–3,98	NA
Tipo de propiedad	Dueño	0,95	0,23–3,86	Ingresos
Tipo de piso	Cemento	0,20	0,03–1,32	Ingresos, tipo de propiedad
Tipo de pared	Cemento	0,96	0,14–6,83	Ingresos, tipo de propiedad
Nivel educativo	Una persona terminó secundaria en el hogar	0,74	0,21–2,64	Vereda
Ingresos	Mayores a un salario mínimo	0,54	0,13–2,22	Nivel educativo
Cerdo	Sí	0,20	0,05–0,74 *	Estación, baño, ingresos, nivel educativo, perro, tenencia de pozo séptico, fuente, tipo de propiedad, vereda
Perro	Sí	0,35	0,10–1,26	Cerdo, estación, baño, ingresos, nivel educativo, tenencia de pozo séptico, tipo de fuente, tipo de propiedad, vereda
Gato	Sí	1,84	0,40–8,46	Tipo de propiedad
Sexo	Femenino	1,56	0,51–4,75	NA
Edad	< 5 años	4,05	1,70–9,68 *	NA
Condiciones médicas	Sí	2,21	0,34–14,47	Edad, género, ingresos, seguro de salud
Seguro de salud	Si	0,59	0,08–4,45	Ingresos, vereda
Tipo de fuente	Lluvia	5,20	0,60-45,18	Cerdo, estación, baño, ingresos, nivel educativo, perro, tenencia de pozo séptico, tipo de propiedad, vereda
	Estanco	11,47	1,27-103,29 *	
	Pozo profundo	16,90	2,45-116,67 *	
	Río	1,00	NA	
Disposición de residuos sólidos	Quema	1,14	0,26–4,95	Tipo de propiedad
	Aire libre	3,97	0,74–21,35	
	Ambos	1,00	NA	
Lavamanos	Sí	0,60	0,06–6,10	Ingresos, tipo de propiedad, baño
Baño	Sí	0,23	0,06–0,96 *	Ingresos, tenencia de pozo séptico, tipo de propiedad
Pozo séptico	Sí	0,55	0,15–1,99	Ingresos, tipo de propiedad
Nivel de riesgo	Inviabilidad sanitaria	1,13	0,13–9,43	Estación, tenencia de lavamanos, tipo de fuente de agua

a RP: razón de prevalencia.

b IC95%: intervalo de confianza del 95%.

c Conjunto de variables para ajustar resultantes del DAG

* Significancia estadística para α = 0,05.

## DISCUSIÓN

Se observan condiciones precarias de saneamiento básico y abastecimiento de agua para consumo humano en las veredas ubicadas en área rural dispersa del Caribe colombiano. Ninguna de las viviendas contaba con servicio de recolección de residuos, alcantarillado y acueducto, tampoco con sistema comunitario de abastecimiento de agua. Sistemas individuales de provisión de agua fueron utilizados desde diferentes tipos de fuentes (agua lluvia, pozo profundo, estanco y/o río), en algunos casos, localizadas a cientos de metros de distancia. Esa situación dificulta el transporte y restringe la cantidad de agua para aprovisionamiento. Estas características coinciden con las de poblaciones periurbanas y rurales en países subdesarrollados reportadas por Razzolini y Günther ^21^ .

Las muestras de agua provenientes desde diversas fuentes de abastecimiento tuvieron diferentes calidades, influenciadas por la naturaleza de estas y los puntos de contaminación circundantes. La alta carga de bacterias fecales fue un factor universal en las muestras de agua. Contaminación que es más probable encontrar en zonas rurales debido a actividades pecuarias ^22^ , defecación al aire libre ^23^ , aseo personal cercano o dentro de la fuente y manipulación, transporte y almacenamiento inadecuado del agua desde la fuente hasta los hogares ^24^^,^^25^ .

La prevalencia de enfermedad diarreica en las veredas El Cascajo y La Delfina fue 7,5% en todas las edades. No obstante, en menores de cinco años, ese valor llegó a 23,5%, lo que es 91,0% mayor a la prevalencia promedio reportada para Colombia (12,3%) ^26^ , pero es similar a las prevalencias promedios reportadas para países de bajos ingresos como Burundi, Camerún, Haití, Nigeria, Uganda y Senegal ^26^ .

Encontramos también una mayor ocurrencia de enfermedad diarreica en estación seca en comparación con la época de lluvia, resultado que concuerda con evidencias reportadas por Wilopo et al. ^27^ y Vila et al ^28^ . Esta asociación puede explicarse por el aumento en el uso de agua de estanco como fuente de abastecimiento durante esta época. La misma fuente también es utilizada como abrevadero de animales, los cuales aportan carga microbiológica al agua.

En contraste, algunos estudios han detectado una mayor frecuencia de enfermedad diarreica en época de lluvias ^25^^,^^29^ , lo cual puede ser explicado por el arrastre de la contaminación circundante a causa de escorrentías. No obstante, para el presente estudio, la disminución de los casos de enfermedad diarreica en época de lluvias pudo estar influenciada por la preferencia de consumir agua lluvia por encima de aquellas que son influenciadas por escorrentías (río y estanco).

En el modelo sin ajustar, ninguna de las fuentes de agua presentó asociación estadísticamente significativa con los casos de enfermedad diarreica. En el modelo ajustado, observamos asociaciones estadísticamente significativas para el agua de estanco y pozo profundo, con una razón de prevalencia que las sugiere como factores de riesgo. Lo anterior refleja un fenómeno de confusión que pudo ser mitigado por el set de variables con el que se ajustó el modelo, en el cual se destacan las variables: estación y vereda. La primera variable podría influenciar la exposición a enfermedad diarreica al favorecer el consumo de agua lluvia en época de altas precipitaciones. Por otra parte, la vereda puede influenciar el acceso a determinados tipos de fuente de acuerdo con la geografía.

La tenencia de baño estuvo asociada con el desenlace pareciendo un factor protector. Aunque las demás variables de saneamiento básico no arrojaron diferencias estadísticamente significativas, observamos menor prevalencia en aquellas viviendas que tenían instalado lavamanos y/o pozo séptico.

La presencia de cerdo resultó ser un factor protector en el modelo ajustado. Algunos estudios exponen que los animales domésticos generalmente son factores de riesgo ^6^ . Sin embargo, consideramos que la tenencia de cerdo podría ser un indicador de mejores condiciones socioeconómicas dentro de las familias encuestadas y tal vez, mejores condiciones y hábitos de higiene.

Futuros estudios que analicen la asociación entre enfermedad diarreica y la tenencia de cerdos podrán contrastar con los resultados observados y determinar si la presencia de cerdos es un factor protector de enfermedad diarreica en lugares con deficientes condiciones de saneamiento básico.

Documentamos elevados valores de IRCA en fuentes de consumo humano. Eso explicaría las fuertes asociaciones encontradas entre algunas de las fuentes de agua y la frecuencia de enfermedad diarreica. De esta forma, nuestros resultados evidencian el impacto de la baja tasa de tratamiento de agua, reflejando el escaso conocimiento que se tiene acerca de los riesgos en la salud bajo estas exposiciones.

Estos resultados deben promover programas locales y regionales a largo plazo para mejorar la calidad de agua en el punto de uso, mediante acciones como la desinfección solar, coagulación con alumbre, desinfección con hipoclorito de sodio o ebullición del agua. Estas intervenciones deben acompañarse de campañas educativas en higiene y saneamiento básico cuyos objetivos sean: a) advertir a la comunidad acerca de los riesgos para la salud del consumo de agua con calidad deficiente y de las condiciones precarias de saneamiento básico; b) capacitar acerca de los cuidados en el transporte, manipulación y almacenamiento de agua; e, c) informar sobre la tenencia responsable de animales domésticos.

Lo anterior debe ser una medida transitoria a soluciones de mayor envergadura que involucre la generación de políticas diferenciales y transversales para el sector rural, que tenga en cuenta de manera integral la problemática de abastecimiento y calidad de agua junto con la adecuada disposición de excretas y residuos. Para garantizar la integración de diferentes sectores, estas políticas podrán incorporar estrategias como: a) incentivos para la creación de empresas sociales que opten por el diseño de tecnologías alternativas a las soluciones convencionales; b) programas educativos en escuelas rurales relacionadas con higiene y saneamiento básico; c) asistencia técnica a la comunidad; y, d) vinculación de la comunidad para que participe de manera activa en el reporte efectivo tanto del control de la calidad de agua como de los casos de enfermedades diarreicas. Todo esto con el fin de generar mejoras sostenibles en la calidad del agua de consumo para el control de las enfermedades diarreicas que afecta principalmente a la población pediátrica.

Este estudio tiene algunas limitaciones. Aunque el período de recuerdo de siete días es comúnmente usado en la práctica para evitar el subregistro de información ^30^ , dicho periodo puede ser muy corto para obtener información representativa sobre la carga de enfermedad diarreica en las familias. Subregistros de casos de enfermedad diarreica pudieron también haberse presentado por parte de la persona que respondía la encuesta, debido al desconocimiento de sintomatología presente en el resto de los individuos o por pudor al revelar dicha información.

Aun cuando se midieron las características fisicoquímicas y microbiológicas del agua en cada uno de los hogares, la calidad de agua resultante no representa la calidad del agua consumida en los siete días anteriores a la encuesta. No obstante, el IRCA calculado señala el alto riesgo que presentó el agua de consumo humano en las dos veredas en el momento del estudio.

Por otro lado, el limitado tamaño de la muestra se reflejó en una precisión limitada y, probablemente, en un poder reducido para identificar otros factores asociados con enfermedad diarreica. Sin embargo, los resultados obtenidos exponen un diagnóstico de la zona estudiada. De igual manera, el diagrama causal elaborado, cuyas implicaciones fueron evaluadas y mostraron consistencia con los datos, puede ser una herramienta conceptual útil para futuras investigaciones que compartan el mismo contexto rural.

Las largas distancias entre viviendas y las difíciles vías de transporte que caracterizan el área rural dispersa no sólo limitan la implementación de servicios de acueducto y de saneamiento básico, sino que también dificultan la logística de este tipo de estudios. Se espera que estos resultados sirvan como evidencia de referencia y facilite el camino a tomadores de decisiones y a otros investigadores, para llevar a cabo estudios complementarios sobre el tema.

A pesar de los esfuerzos realizados en materia de agua potable y de mejoramiento de las condiciones de vida de la población más vulnerable en Colombia, en área rural dispersa aún se observan condiciones deficientes relacionadas con abastecimiento de agua, higiene y saneamiento básico. Urge un diseño efectivo de políticas que contribuyan al mejoramiento de condiciones ambientales y saneamiento en viviendas, y que reduzcan la prevalencia de enfermedad diarreica, especialmente en niños menores de cinco años.
